# Measuring religiosity: comparison of single-item measures with validated scales in a UK cohort study (ALSPAC)

**DOI:** 10.12688/wellcomeopenres.23943.1

**Published:** 2025-04-18

**Authors:** Jimmy Morgan, Isaac Halstead, Jean Golding, Kate Northstone, Daniel Major-Smith

**Affiliations:** 1 Centre for Academic Child Health, Population Health Sciences, Bristol Medical School, University of Bristol, Bristol, England, UK; 2 Population Health Sciences, Bristol Medical School, University of Bristol, Bristol, England, UK; 3 Department for the Study of Religion, School of Culture and Society, Aarhus University, Aarhus, Denmark

**Keywords:** ALSPAC, factor analysis, religion, religiosity, measurement

## Abstract

**Background:**

Many studies use single-item variables to measure religiosity, such as religious belief, identity or service attendance. However, there are many different hypothesised dimensions of religiosity and it is often unclear how these single-item measures may map onto these theorised constructs. ALSPAC (Avon Longitudinal Study of Parents and Children) primarily relied on single-items to measure religiosity, but more recently has included validated questionnaires (DUREL [Duke University Religion Index] and I/EMSS [Intrinsic/Extrinsic Measurement: I/E-Revised and Single-Item Scales]). This paper aims to: i) confirm whether the validated measures work as intended in ALSPAC; and ii) understand which religiosity dimensions the single-item measures relate to.

**Methods:**

Twenty religiosity questions were asked to ALSPAC offspring and parents approximately 28 years after the offspring’s birth. We used three exploratory factor analyses to assess how the different items related to one another on: i) the pre-validated DUREL and I/EMSS measures to ensure they function as intended; ii) all 20 religiosity measures; and iii) the pre-validated measures and the single-item measures also used at previous ALSPAC timepoints (13 measures).

**Results:**

The first factor analysis showed that, beyond a single religiosity factor, these pre-validated items did not always work as intended. For instance, intrinsic religiosity items loaded together, but extrinsic religiosity items were often separated. The second and third analyses showed that single-item measures did not relate well to hypothesised dimensions of religion but did form two broad factors of belief-based and behaviour-based items. Results were broadly comparable across both ALSPAC generations.

**Conclusions:**

These results show that pre-validated measures of religiosity do not always behave as expected in ALSPAC, while the single-item measures do not easily map onto specific dimensions of religiosity. These results will help researchers better understand the ALSPAC religiosity data and contribute to the debate on the factor structure of religion more broadly.

## Plain Language summary

A person’s religiosity encompasses a huge range of factors and considerations, this makes measuring religiosity very difficult. To avoid over-burdening participants of questionnaires, difficult choices have to be made on which questions are most important to include. In general, researchers try to include as many aspects of religion as possible. Some of the main categories of religious measures include whether they are done on your own or in public and these are further categorised into whether they are internal beliefs or whether they include a behaviour.

We aimed to find how our measures of religiosity at ALSPAC fit with existing ideas on how to categorise religiosity. We did this by using ‘factor analysis’, this is a technique that helps identify the underlying patterns between measures to help group them together into ‘factors’. The hope was that these factors would align with existing ideas of how religious measures should be categorised.

However, despite using some pre-validated measures to help calibrate the analysis the measures did not align as well as hoped with existing literature. Measures relating to more belief based characteristics separated from behaviour based measures but most other results do not lend themselves to easy interpretation.

## Background

It has been argued that religion research needs to take a “step back and consider what it is that we are actually measuring” ^1^. While there will likely be strong correlations between variables measuring religiosity ^2^, there may also be different clusters of religiosity items;, many measures have been developed to capture these ^3, 4^. They are often categorised as intrinsic or extrinsic and maybe further divided into cognitive, behavioural, and affective dimensions ^5^. However, for expedience, space and avoiding participant over-burden, many studies – especially large-scale surveys and longitudinal population-based studies – often rely on single-item measures such as religious attendance ^6^, importance of religion, and religious affiliation ^2, 7^. Whilst these measures provide some predictive validity when it comes to wider theoretical constructs, and likely correlate with religious dimensions, their exact relation with these constructs is often unclear.

Without evidence showing how measures relate to each other, researchers may also not understand how to best interpret results from various measures of religiosity in their models ^2^ and different measures of religiosity may have different relationships with study outcomes. For example, religious service attendance may be related closely to extrinsic religiosity and behavioural dimensions but is also an indicator of religious salience, a combination of two dimensions hypothesized in Pearce, Hayward, and Pearlman, 2017.

The Avon Longitudinal Study of Parents and Children (ALSPAC) is a birth cohort UK based and consists of a large cohort of parents and offspring, who have completed a wide range of religion measures across multiple time-points. Most prior ALSPAC data collections on religion included only single-item measures (Iles-Caven *et al.*, 2019). Whilst relevant to understanding religiosity in this cohort, these measures are difficult to categorise into previously hypothesized dimensions. For example, how do belief in God, regular religious attendance and praying when not in trouble relate to intrinsic or extrinsic dimensions of religiosity? In short, ALSPAC has lots of repeated religiosity data but it is not clear how these variables relate to validated religiosity dimensions identified in the literature, or how well they relate to one another.

When the study offspring were approximately 28 years old a broader range of religion questions were asked of offspring and their parents, including these previous single-item measures and items taken from validated scales, including the Duke University Religion Index (DUREL) and Intrinsic/Extrinsic Measurement Scale (I/EMSS; Table 1). This paper aims to examine how the single-item measures used in ALSPAC relate to hypothesized dimensions of religiosity, with a specific focus on intrinsic and extrinsic religiosity.

**Table 1.** The questions used in each of the factor analyses as well as frequencies at the age 28 timepoint for all three cohorts (G0 = Generation-0 [i.e., parental generation]; G1 = Generation-1 [i.e., offspring generation]). Analysis 1 is only the pre-validated scales, Analysis 2 is all measures, and Analysis 3 is the pre-validated measures and the single item measures used at previous ALSPAC timepoints.

Question	Response				Analysis 1	Analysis 2	Analysis 3
	Responses	Frequency (Percentage)					
		G0 Mothers	G0 Partners	G1			
Do you believe in God or in some divine power?	Yes	1,989 (43.6%)	632 (30.0%)	704 (16.8%)		✓	✓
	Not Sure	1,355 (29.7%)	516 (24.5%)	1,103 (26.4%)			
	No	1,219 (26.7%)	958 (45.5%)	2,377 (56.8%)			
	*Missing*	*9,385 (67.3%)*	*11,842 (84.9%)*	*9,764 (70.0*% *)*			
Do you feel that God (or some divine power) has helped you at any time?	Yes	1,589 (34.9%)	491 (23.5%)	652 (15.6%)		✓	✓
	Not Sure	1,159 (25.5%)	404 (19.3%)	764 (18.3%)			
	No	1,804 (39.6%)	1.199 (57.3%)	2,761 (66.1%)			
	*Missing*	*9,396 (67.4*% *)*	*11,854 (85.0*% *)*	*9,771 (70.1*% *)*			
Would you appeal to God for help if you were in trouble?	Yes	2,214 (48.7%)	643 (30.7%)	877 (21.0%)		✓	✓
	Not Sure	880 (19.4%)	394 (18.8%)	755 (18.1%)			
	No	1,453 (32.0%)	1,061 (50.6%)	2,541 (60.9%)			
	*Missing*	*9,401 (67.4*% *)*	*11,850 (85.0*% *)*	*9,775 (70.1*% *)*			
Do you ‘pray’ even if not in trouble?	Yes	1,532 (33.9%)	426 (20.4%)	481 (11.5%)		✓	
	Not Sure	312 (6.9%)	125 (6.0%)	267 (6.4%)			
	No	2,679 (59.2%)	1,542 (73.7%)	3,425 (82.1%)			
	*Missing*	*9,425 (67.6*% *)*	*11,855 (85.0*% *)*	*9,775 (70.1*% *)*			
Do you go to a place of worship? (DUREL; Organisational religiosity subscale)	At least once a week	404 (9.0%)	163 (7.8%)	126 (3.0%)	✓	✓	✓
	At least once a month	198 (4.4%)	79 (3.8%)	63 (1.5%)			
	At least once a year	344 (7.6%)	154 (7.4%)	310 (7.5%)			
	Occasionally	1,317 (29.2%)	478 (22.9%)	547 (13.2%)			
	Not at all	2,252 (49.9%)	1,215 (58.2%)	3,104 (74.8%)			
	*Missing*	*9,433 (67.6*% *)*	*11,859 (85.0*% *)*	*9,798 (70.4*% *)*			
Do you obtain help and support from leaders of your religious group?	Yes	410 (9.1%)	174 (8.4%)	148 (3.6%)		✓	✓
	No	4,080 (90.9%)	1,907 (91.6%)	3,976 (96.4%)			
	*Missing*	*9,458 (67.8*% *)*	*11,867 (85. 1*% *)*	*9,824 (70.4*% *)*			
Do you obtain help and support from other members of your religious group?	Yes	507 (11.4%)	196 (9.5%)	200 (4.9%)		✓	✓
	No	3,934 (88.6%)	1,864 (90.5%)	3,917 (95.1%)			
	*Missing*	*9,507 (68.2*% *)*	*11,888 (85.2*% *)*	*9,831 (70.5*% *)*			
Do you obtain help and support from members of other religious groups?	Yes	64 (1.5%)	27 (1.4%)	19 (0.5%)		✓	✓
	No	4,167 (98.5%)	1,942 (98.6%)	3913 (99.5%)			
	*Missing*	*9,717 (69.7*% *)*	*11,979 (85.9*% *)*	*10,016 (71.8*% *)*			
Do you obtain help and support from leaders of other religious groups?	Yes	106 (2.5%)	*42 (2.2*% *)*	*48 (1.2*% *)*		✓	
	No	4,078 (97.5%)	*1,905 (97.8*% *)*	*3,866 (98.8*% *)*			
	*Missing*	*9,764 (70.0*% *)*	*12,001 (86.0*% *)*	*10,034 (71.9*% *)*			
How often do you spend time in private religious activities, such as prayer, meditation, or holy scripture study? (DUREL; Non- organisational religious activity subscale)	More than once a day	126 (2.8%)	55 (2.7%)	46 (1.1%)	✓	✓	✓
	Daily	344 (7.7%)	96 (4.7%)	95 (2.3%)			
	2+ times a week	254 (5.7%)	78 (3.8%)	97 (2.4%)			
	Once a week	121 (2.7%)	36 (1.8%)	47 (1.1%)			
	Few times a month	277 (6.2%)	80 (3.9%)	138 (3.4%)			
	Rarely or Never	3,364 (75.0%)	1,711 (83.2%)	3,685 (89.7%)			
	*Missing*	*9,462 (67.8*% *)*	*11,892 (85.3*% *)*	*9,840 (70.6*% *)*			
How often do you listen to/watch religious programming on the radio/ television/social media?	Daily	40 (0.9%)	17 (0.8%)	23 (0.6%)		✓	
	Several times a week	88 (2.0%)	32 (1.5%)	40 (1.0%)			
	Several times a month	126 (2.8%)	46 (2.2%)	38 (0.9%)			
	Occasionally	1,353 (29.9%)	528 (25.2%)	292 (7.1%)			
	Never	2,917 (64.5%)	1,470 (70.2%)	3,739 (90.5%)			
	*Missing*	*9,424 (67.6*% *)*	*11,855 (85.0*% *)*	*9,816 (70.4*% *)*			
How often do you read religious related texts or publications?	Daily	208 (4.6%)	76 (3.6%)	55 (1.3%)		✓	
	Several times a week	118 (2.6%)	46 (2.2%)	71 (1.7%)			
	Several times a month	98 (2.2%)	45 (2.2%)	48 (1.2%)			
	Occasionally	613 (13.6%)	247 (11.8%)	266 (6.4%)			
	Never	3,485 (77.1%)	1,682 (80.3%)	3,691 (89.4%)			
	*Missing*	*9,426 (67.6*% *)*	*11,852 (85.0*% *)*	*9,817 (70.4*% *)*			
In my life, I experience the Presence of the Divine (DUREL; intrinsic religiosity subscale)	Definitely true of me	484 (10.8%)	153 (7.3%)	158 (3.8%)	✓	✓	✓
	Tends to be true of me	487 (10.9%)	150 (7.2%)	192 (4.7%)			
	Unsure	763 (17.0%)	250 (12.0%)	415 (10.1%)			
	Tends not to be true of me	390 (8.7%)	162 (7.8%)	235 (5.7%)			
	Definitely not true of me	2,364 (52.7%)	1,371 (65.7%)	3,126 (75.8%)			
	*Missing*	*9,460 (67.8*% *)*	*11,862 (85.0*% *)*	*9,822 (70.4*% *)*			
My religious beliefs are what really lie behind my whole approach to life. (DUREL; intrinsic religiosity subscale)	Definitely true of me	448 (10.0%)	153 (7.4%)	143 (3.5%)	✓	✓	✓
	Tends to be true of me	694 (15.5%)	248 (11.9%)	211 (5.1%)			
	Unsure	491 (11.0%)	139 (6.7%)	269 (6.5%)			
	Tends not to be true of me	470 (10.5%)	190 (9.1%)	236 (5.7%)			
	Definitely not true of me	2,382 (53.1%)	1,351 (65.0%)	3,262 (79.2%)			
	*Missing*	*9,463 (67.8*% *)*	*11,867 (85.1*% *)*	*9,827 (70.5*% *)*			
I try hard to carry my religion over into all other dealings in life. (DUREL; intrinsic religiosity subscale)	Strongly Agree	400 (9.0%)	145 (7.0%)	118 (2.9%)	✓	✓	✓
	Mildly Agree	633 (14.2%)	211 (7.8%)	198 (4.8%)			
	Not Sure	481 (10.8%)	142 (6.8%)	205 (5.0%)			
	Mildly Disagree	434 (9.7%)	162 (7.8%)	208 (5.1%)			
	Strongly Disagree	2,522 (56.4%)	1,419 (68.3%)	3,392 (82.3%)			
	*Missing*	*9,478 (68.0*% *)*	*11,869 (85.1*% *)*	*9,827 (70.5*% *)*			
I attend a place of worship mainly because it helps me make friends. (I/EMSS; socially extrinsic subscale)	Strongly Agree	85 (1.9%)	17 (0.8%)	47 (1.1%)	✓	✓	✓
	Mildly Agree	336 (7.5%)	124 (6.0%)	126 (3.1%)			
	Not Sure	148 (3.3%)	75 (3.6%)	51 (1.2%)			
	Mildly Disagree	297 (6.6%)	121 (5.8%)	100 (2.4%)			
	Strongly Disagree	3,611 (80.7%)	1,742 (83.8%)	3,797 (92.1%)			
	*Missing*	*9,471 (67.9*% *)*	*11,869 (85.1*% *)*	*9,827 (70.5*% *)*			
I pray mainly to gain relief and protection. (I/EMSS; personally extrinsic subscale)	Strongly Agree	206 (4.6%)	35 (1.7%)	59 (1.4%)	✓	✓	✓
	Mildly Agree	713 (16.0%)	165 (7.9%)	302 (7.4%)			
	Not Sure	379 (8.5%)	121 (5.8%)	162 (3.9%)			
	Mildly Disagree	329 (7.4%)	142 (6.8%)	115 (2.8%)			
	Strongly Disagree	2,836 (63.5%)	1,614 (77.7%)	3,470 (84.5%)			
	*Missing*	*9,485 (68.0*% *)*	*11,871 (85.1*% *)*	*9,840 (70.6*% *)*			
To what extent do you consider yourself a religious person?	Very	108 (2.4%)	44 (2.1%)	55 (1.3%)		✓	
	Moderately	619 (13.8%)	243 (11.6%)	182 (4.4%)			
	Slightly	1,491 (33.1%)	490 (23.4%)	667 (16.1%)			
	Not at all	2,285 (50.7%)	1,316 (62.9%)	3,228 (78.1%)			
	*Missing*	*9,445 (67.7*% *)*	*11,855 (85.0*% *)*	*9,816 (70.4*% *)*			
To what extent do you consider yourself a spiritual person?	Very	355 (7.9%)	94 (4.5%)	172 (4.2%)		✓	
	Moderately	905 (20.1%)	331 (15.9%)	459 (11.1%)			
	Slightly	1,326 (29.4%)	424 (20.3%)	1,099 (26.6%)			
	Not at all	1,919 (42.6%)	1,239 (59.3%)	2,398 (58.1%)			
	*Missing*	*9,443 (67.7*% *)*	*11,860 (85.0*% *)*	*9,820 (70.4*% *)*			
How important to you is religion or spirituality?	Highly	642 (14.3%)	216 (10.3%)	235 (5.7%)		✓	
	Moderately	758 (16.8%)	270 (12.9%)	362 (8.8%)			
	Slightly	1,348 (29.9%)	457 (21.8%)	901 (21.8%)			
	Not at all	1,757 (39.0%)	1,152 (55.0%)	2,630 (63.7%)			
	*Missing*	*9,443 (67.7*% *)*	*11,853 (85.0*% *)*	*9,820 (70.4*% *)*			

## Methods

### Participants

Pregnant women resident in Avon, UK with expected delivery dates between 1 ^st^ April 1991 and 31 ^st^ December 1992 were invited to participate. The initial number of pregnancies enrolled was 14,541, of which there were 14,676 foetuses, resulting in 14,062 live births and 13,988 children alive at 1 year of age; after additional recruitment from age 7, there were a total of 14,833 unique mothers enrolled and 14,901 children alive at 1 year of age ^8– 12^. 12,113 of the mother’s partners have also been in contact with the study, with 3,807 currently enrolled. After removing pregnancies that did not result in a live birth, excluding participants who were enrolled at a stage other than during pregnancy, and dropping observations for participants who had withdrawn consent for their data to be used, there were a total of 13,948 G0 (Generation-0) mothers and 11,737 associated partners. A possible 13,948 G1 (Generation-1) were included, consisting of all children alive at one year of age who had not withdrawn consent.

### Religiosity variables

When G1 were 28 years of age, three sets of religiosity questions were asked to G0 mothers, partners and G1 ( Table 1): i) single-item variables previously asked of parents (Iles-Caven *et al.*, 2019); ii) single-item measures only asked at age 28; and iii) questions taken from validated religiosity scales ^13^, including the DUREL ^14^ and the I/EMSS ^15^. There were 7 pre-validated measures of religiosity, inlcuding three measures of intrinsic religiosity (experiences presence of divine, beliefs influence whole approach to life and carries religious ideas into all aspects of life), two measures of extrinsic religiosity (attends a place of worship to help make friends and prays mainly for relief and protection), one measure of organisational religious activity (frequency attends a place of worship; although this was also asked at previous ALSPAC time-points) and one measure of non-organisational religious activity (amount of time spent in private religious activities e.g., prayer).

### Analysis

Three exploratory factor analyses were performed on each cohort. The first was conducted using the 7 pre-validated religious measures take from the DUREL and I/EMSS, and was undertaken to ensure that the previously-validated items adhered to the factor structure and reflect separate dimensions as intended. The second analysis included all 20 of the religiosity measures in Table 1, and was intended to provide a broad overview of the underlying factor structure of these data and how the 13 single-item measures load onto the pre-validated measures to help interpret these measures in future work. The third analysis is similar to the second, but focuses specifically on comparing the 7 single-item measures used repeated by ALSPAC since the start of the study to see how these load onto the pre-validated measures; this will not only help us to interpret these measures in light of these pre-validated scales (as per analysis 2), but also help interpret analyses using these ALSPAC single-item measures measured prior to age 28 (assuming the relationships at age 28 will be similar to those at prior time-points).

For all analyses, parallel analyses and scree plots were used to determine the optimum number of factors to extract; where these differed, we compared results using a range of factor solutions ^16, 17^. Exploratory factor analysis was then carried out based on the number of factors suggested, with promax rotation applied to allow for factor scores to be correlated with one another. Any item loading onto a factor at more than 0.6 was considered ‘high’ and any loading between 0.3 and 0.6 was considered ‘ok’. To measure the model fit we used three different measures: the Tucker-Lewis Index (TLI), root mean square of approximation (RMSEA) and the Bayesian Information Criterion (BIC). The TLI is an incremental fit index that compares chi-squared values of the hypothesised and null models where the higher the value on a scale of 0 to 1 indicates better fit ^18, 19^. The RMSEA is an absolute fit index that assesses the discrepancy between the covariance matrices of the hypothesised and perfect models, meaning the lower the number on a scale of 0 to 1 indicates better fit ^19^. These indices are traditionally used with cut-off points to indicate ‘good’ models, however, we will be using them purely as a value to indicate the goodness of model fit and compare between models ^19, 20^. The BIC is a measure of relative model fit, with lower values indicating greater fit, with a penalty for over-fitting. As all religiosity items were ordered categorical variables, polychoric correlations were used throughout. A sensitivity analysis was also conducted on all cohorts using all 20 religiosity variables (analysis 2, above), treating the items as continuous rather than ordinal to test the robustness of our results. Analyses were conducted in R (v4.1.1; ^21^) using the packages *haven, psych, GPArotation,* and *tidyverse* ^22– 25^.

## Results

**Figure 1.**
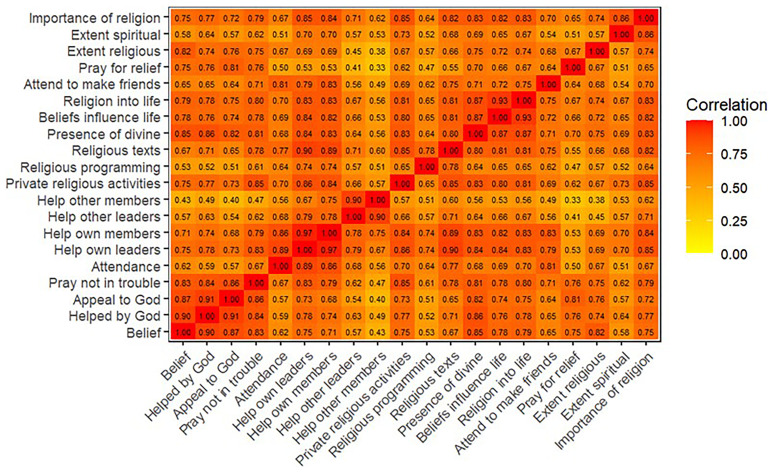
Heatmap of the polychoric correlation matrix of all 20 religiosity variables used in analysis for G0 mothers.

**Figure 2.**
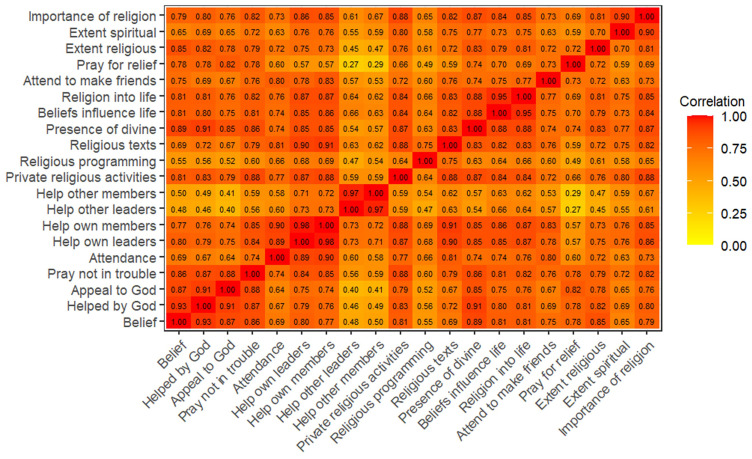
Heatmap of the polychoric correlation matrix of all 20 religiosity variables used in analysis for G0 partners.

**Figure 3.**
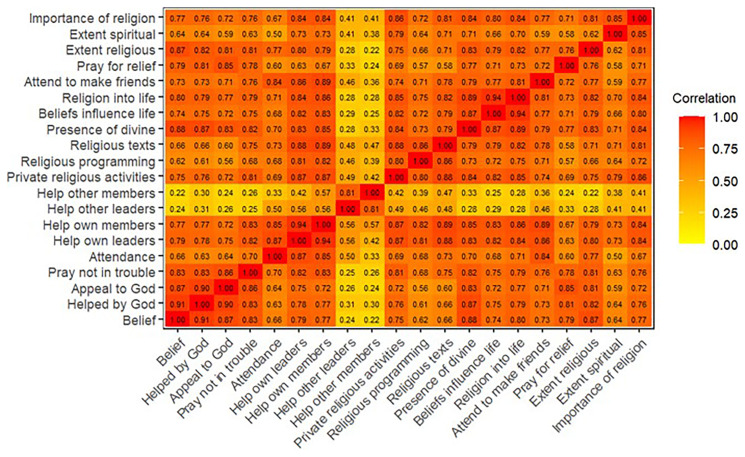
Heatmap of the polychoric correlation matrix of all 20 religiosity variables used in analysis for G1.

Descriptive statistics are presented in Table 1 (interpretation of these has been discussed in-depth previously ^26^. Heatplots of the polychoric correlation matrices are displayed in Figure 1 (G0 mothers), Figure 2 (G0 partners) and Figure 3 (G1), and indicate that most of the religion variables are highly-correlated, albeit with some variables being more correlated than others.

### Analysis 1: Pre-validated measures

The parallel analysis (Figure S1) suggested 4 factors for the mothers’ cohort, although visual inspection of the scree plot suggested only 1 major factor, with little improvement after 2 or more. We therefore completed an exploratory factor analysis for 1 to 4 factors to compare the model fits and interpretability. A one factor solution had high loadings (>0.7) for all items, but had the worst measures of model fit. Increasing the number of loadings marginally improved model fit ( Table 2), with more complex models separating most variables other than a core group of intrinsic religiosity measures ( Table 3). In models with two or more factors, the extrinsic religiosity variables (attendance to make friends [socially extrinsic] and pray for relief and protection [personally extrinsic]) loaded onto different factors. There appeared to be model fit issues for the four-factor solution. See Figure S1 and Tables S1–S4 for full results. These analyses were repeated for the partner’s and G1 cohorts, with broadly similar results (Figures S2–3 and Tables 2, 3 & S5–S12).

**Table 2.** Measure of model fit and proportions of variance explained for the exploratory factor analysis 1 (for the 7 pre-validated religiosity measures). TLI = Tucker-Lewis Index, RMSEA = Root Mean Square Error of Approximation, BIC = Bayesian Information Criterion. Mothers sample size = 4,272. Partners sample size = 1,998, G1 sample size = 4,034.

G0 Mothers								
No. of Factors	Proportion of Variance Explained per Factor (%)				Total Proportion of Variance Explained (%)	TLI	RMSEA	BIC
	First	Second	Third	Fourth				
One	75	-	-	-	75	0.87	0.22	2776.9
Two	56	25	-	-	81	0.90	0.17	980.7
Three	48	24	15	-	87	0.90	0.20	475.4
Four	49	14	14	14	91	1.01	NA	NA
G0 Partners								
No. of Factors	Proportion of Variance Explained per Factor (%)				Total Proportion of Variance Explained (%)	TLI	RMSEA	BIC
	First	Second	Third	Fourth				
One	78	-	-	-	78	0.86	0.24	1563.2
Two	61	24	-	-	85	0.87	0.23	803.2
Three	48	24	17	-	89	0.81	0.29	476.3
Four	41	16	24	10	91	1.01	NA	NA
G1								
No. of Factors	Proportion of Variance Explained per Factor (%)				Total Proportion of Variance Explained (%)	TLI	RMSEA	BIC
	First	Second	Third	Fourth				
One	78	-	-	-	78	0.86	0.24	3254.1
Two	54	29	-	-	83	0.93	0.18	952.3
Three	50	23	16	-	89	0.92	0.18	366.2
Four	39	23	17	14	93	1.01	NA	NA

**Table 3.** Summary of results for analysis 1 (for the 7 pre-validated religiosity measures). Green indicates best loading for each item. Orange indicates item loaded onto more than one factor similarly or has weak loadings for all factors. Green indicates a loading of greater than 0.6, and orange greater than 0.3. Mothers sample size = 4,272. Partners sample size = 1,998. G1 sample size = 4,034.

	One factor	Two factors		Three factors			Four factors			
G0 mothers	1	1	2	1	2	3	1	2	3	4
Attendance (organisational religiosity)	0.79	-0.05	0.99	0.03	-0.09	0.96	-0.02	0.93	-0.09	0.08
Private religious activities (Non-organisational religiosity)	0.87	0.72	0.19	0.72	-0.01	0.19	0.15	0.07	0.00	0.80
Experience presence of divine (Intrinsic subscale)	0.92	0.92	0.03	0.83	0.09	0.04	0.61	0.05	0.12	0.22
Beliefs influence whole life (Intrinsic subscale)	0.93	0.94	0.01	0.95	0.00	0.00	0.95	0.03	-0.01	0.00
Religious ideas carried into life (Intrinsic subscale)	0.94	0.91	0.05	0.92	0.00	0.04	0.95	0.07	-0.02	-0.03
Attendance to make friends (Socially extrinsic subscale)	0.84	0.29	0.63	0.11	0.15	0.69	0.13	0.75	0.14	-0.07
Pray for relief and protection (Personally extrinsic subscale)	0.73	0.67	0.08	0.03	0.98	-0.02	0.00	-0.01	1.00	0.00
G0 partners	1	1	2	1	2	3	1	2	3	4
Attendance (organisational religiosity)	0.84	0.53	0.37	0.09	-0.08	0.93	0.05	-0.08	0.81	0.18
Private religious activities (Non-organisational religiosity)	0.90	0.89	0.03	0.72	-0.03	0.24	0.43	0.00	0.16	0.47
Experience presence of divine (Intrinsic subscale)	0.93	0.95	-0.01	0.81	0.13	0.04	0.59	0.16	0.01	0.28
Beliefs influence whole life (Intrinsic subscale)	0.94	0.96	-0.01	0.95	0.02	0.00	0.95	0.00	0.04	0.00
Religious ideas carried into life (Intrinsic subscale)	0.95	0.93	0.03	0.90	0.00	0.07	0.92	-0.02	0.11	-0.02
Attendance to make friends (Socially extrinsic subscale)	0.85	-0.06	1.11	0.10	0.23	0.62	0.11	0.17	0.76	-0.10
Pray for relief and protection (Personally extrinsic subscale)	0.77	0.53	0.28	0.04	0.97	-0.01	0.01	0.96	0.02	0.01
G1	1	1	2	1	2	3	1	2	3	4
Attendance (organisational religiosity)	0.80	0.01	0.89	-0.02	1.02	-0.05	-0.06	1.02	-0.05	0.04
Private religious activities (Non-organisational religiosity)	0.89	0.75	0.17	0.77	0.13	0.01	0.18	0.05	-0.01	0.81
Experience presence of divine (Intrinsic subscale)	0.93	0.85	0.11	0.77	0.04	0.16	0.57	0.06	0.18	0.19
Beliefs influence whole life (Intrinsic subscale)	0.92	0.95	0.00	0.98	-0.03	-0.01	0.99	-0.02	-0.01	0.00
Religious ideas carried into life (Intrinsic subscale)	0.95	0.92	0.06	0.98	0.03	-0.03	0.96	0.05	-0.03	0.01
Attendance to make friends (Socially extrinsic subscale)	0.89	0.15	0.82	0.25	0.59	0.14	0.25	0.62	0.14	-0.03
Pray for relief and protection (Personally extrinsic subscale)	0.80	0.56	0.27	0.03	-0.01	0.98	0.00	-0.01	1.01	-0.01

Given the abnormal model fit values and some items loading onto individual factors, there is a high probability that these results are over-extracted/overfitted and unreliable, especially for three- or four-factor solutions. Nonetheless, some conclusions regarding the factor structure of these variables can be drawn: 1) all of these validated religion variables are highly-correlated; 2) despite the risk of over-extracting/overfitting, some measures do appear more correlated than others; 3) in some instances these factors map onto proposed religious dimensions (e.g., intrinsic religiosity), while for others they do not (e.g., the two measures of extrinsic religiosity appear more correlated with other aspects of RSBB than with each other, especially for ‘attends religious services to make friends’ and frequency of religious service attendance).

### Analysis 2: All measures

The parallel analysis for G0 mothers using all 20 applicable religious items ( Table 1) suggested 6 factors, while the scree plot only suggested 1 or 2 (Figure S4). We therefore conducted exploratory factor analyses for 1 to 6 factors to compare the model fits and interpretability. Here we focus on the 1, 2 and 6 factor solutions (full results of all models in Tables S13–S19) which explained 71%, 78% and 87% of the total variance, respectively ( Table 4 for factor loadings). In the two-factor solution the division was largely between religious belief (e.g., belief in God, appeal to God if in trouble, etc.) and religious behaviours (e.g., religious service attendance, obtaining help/support from religious others, etc.), with others equally-weighted to both (e.g., intrinsic religiosity, importance of religion/spirituality, etc.). In the six-factor solution, religious belief and behaviour remained as the two main factors, but with many of the other previously equally-weighted variables now forming their own small factors (e.g., extent spiritual and importance of religion; 2 of the 3 intrinsic religiosity variables; obtains help from members and leaders of other religious groups (10% variation) and; prays for relief/protection (but weak loading [0.56]).

**Table 4.** Summary of results for analysis 2 for mothers (for all measures). Green indicates best loading for each item. Orange indicates item loaded onto more than one factor similarly or does not meet the threshold. Green indicates a loading of greater than 0.6, and orange greater than 0.3. Mothers sample size = 3,802. Partners sample size = 1,781. G1 sample size = 3,751.

	One factor	Two factors		Six factors					
Belief	0.86	0.96	-0.04	0.98	0.00	-0.09	0.00	0.04	-0.01
Feel God has helped you	0.87	0.89	0.04	0.97	-0.11	0.04	0.13	-0.05	0.01
Appeal to God if in trouble	0.83	1.04	-0.15	1.02	-0.03	-0.03	0.00	-0.04	0.09
Pray when not in trouble	0.89	0.77	0.18	0.64	0.22	0.09	-0.02	0.06	0.06
Attendance	0.80	0.11	0.75	0.05	1.11	-0.13	0.07	-0.21	-0.02
Help from own leaders	0.95	0.17	0.84	0.16	0.64	0.10	0.09	0.03	-0.18
Help from own members	0.93	0.08	0.92	0.02	0.65	0.14	0.17	0.06	-0.08
Help from other members	0.76	-0.14	0.96	0.14	0.04	-0.08	0.89	0.05	-0.02
Help from other leaders	0.64	-0.30	1.00	-0.10	0.07	0.08	0.93	-0.03	0.07
Private religious activities (Non-organisational religiosity)	0.90	0.45	0.51	0.25	0.20	0.34	-0.01	0.20	-0.05
Religious programming	0.72	0.11	0.66	-0.15	0.50	0.00	0.04	0.42	0.04
Read religious texts	0.90	0.19	0.76	-0.07	0.48	0.15	0.01	0.42	-0.04
Experience presence of divine (Intrinsic subscale)	0.92	0.67	0.31	0.52	0.04	0.06	0.02	0.37	-0.02
Beliefs influence whole life (Intrinsic subscale)	0.90	0.54	0.42	0.30	0.10	-0.06	0.01	0.65	0.01
Religious ideas carried into life (Intrinsic subscale)	0.91	0.54	0.43	0.30	0.11	-0.02	0.02	0.60	0.03
Attendance to make friends (Socially extrinsic subscale)	0.81	0.38	0.48	0.13	0.84	-0.03	-0.03	0.02	0.18
Pray for relief and protection (Personally extrinsic subscale)	0.72	0.99	-0.22	0.78	0.01	0.04	0.07	0.04	0.56
Extent to which religious	0.80	0.80	0.05	0.60	0.34	0.07	-0.19	0.02	0.05
Extent to which spiritual	0.74	0.31	0.48	0.00	-0.07	1.05	0.01	-0.13	0.01
Importance	0.91	0.44	0.53	0.16	0.08	0.74	0.05	0.03	0.05

There was therefore little evidence that these single-item measures consistently loaded with hypothesised dimensions from pre-validated scales. Across all models, model fit values were poor, with lower fit for more complex models (Table S19). Similar patterns were again reported for the G0 partners and G1 (full results in Figures S5–S6 and Tables S20–S32).

Overall, it appears that in the ALSPAC cohorts there is little evidence for a clear relationship between the single-item religiosity variables and the pre-validated variables for specific religious dimensions, beyond a single broader ‘religiosity’ factor. That is, both extrinsic religiosity variables were consistently separate from one another, while the intrinsic religiosity variables were either in separate factors from the other variables or equally weighted across the two main factors. However, there was some consistency between solutions and cohorts with some factors loosely based on religious beliefs and behaviours. Sensitivity analyses repeating the above analyses for all cohorts using Pearson, rather than polychoric, correlations, found comparable patterns of factor structures, albeit with lower proportions of variance explained and better model fit values (Tables S33–S52).

### Analysis 3: Excluding new single-item measures

The third analysis conducted used only the pre-validated measures from DUREL and I/EMSS and the ALSPAC measures that were collected at repeated timepoints. This consisted of 13 applicable items for analysis (see Table 1). These results were broadly similar to those above, so not repeated here; that is, the pre-validated variables do not reliably or consistently load onto specific individual factors, beyond a general ‘religiosity’ factor, while multi-factor solutions suggest a broad divide between religious beliefs and behaviours. For full results see supplementary Figures S7–S9 and Tables S53–S67.

## Discussion

The results show that the pre-validated measures of religiosity behave somewhat as expected but not fully. Intrinsic items load onto separate factors, but socially- and personally-extrinsic items rarely load together. The ALSPAC-created single-items also do not appear to fit neatly onto these theoretical dimensions, Beyond a single ‘religiosity’ dimension, there was a broad split between religious belief (e.g. belief in God and would appeal to God if in trouble) and religious behaviour (e.g. service attendance and help from religious others) into two factors, with hypothetical religiosity dimensions either split into different factors (personally-extrinsic with belief vs socially-extrinsic with behaviour) or approximately equally-weighted across both or forming separate factors (depending on the number of factors; as with intrinsic religiosity). Results were similar across all three ALSPAC cohorts examined.

The results of these factor analyses do not lend themselves easily to interpretation and suggest that there is substantial difficulty in grouping these individual questions with wider previously-hypothesised religiosity dimensions, beyond a single ‘religiosity’ factor. Researchers using these data cannot therefore assume that, e.g., praying even when not in trouble reflects an intrinsically religious orientation. Nonetheless, these results will help users of these data understand the structure and the relations – or lack thereof – between these single-item religiosity variables and wider religiosity dimensions and constructs.

There are numerous limitations to this study – the biggest of which being the use of only seven pre-validated items across only two scales covering only four hypothesised dimensions. This means that we may have missed out other potentially important dimensions including behavioural, cognitive and salience ^3^. Most of the four pre-validated measures also had a small number of questions to capture the dimension. Given the nature of secondary data, these issues were unavoidable and likely to be common in large scale population-based studies where a balance has to be struck between breadth, depth and participant burden. Nonetheless, even having these small number of items from pre-validated for comparison purposes is an improvement over many studies which focus only on single-item measures without validation.

Many model, especially for the more complex models in analyses 2 and 3, had poor fit values. This may be due to the large number of highly-correlated skewed ordinal variables when using polychoric factor analysis, which can impact model fit values ^27, 28^. However, as the factor solutions for these polychoric models were comparable to those using Pearson correlations (which had better model fit values), we believe that our results are robust.

Another limitation to this study is generalisability, as this study was conducted in the UK on a largely white, Christian/non-religious cohort. The pre-validated religiosity items were validated in US cohorts and the ALSPAC single item measures were developed by University of Bristol researchers with the UK cohort in mind ^15, 29^. We therefore cannot assume these results will replicate in other populations. This also highlights the issue of measurement invariance, as the pre-validated measures were developed in the USA they were developed for more religious cohorts, as the UK is quite irreligious compared to the USA and many non-western countries. This could cause a lack of compatibility between results in different cohorts vs ALSPAC ^30– 33^.

Despite these limitations, this paper has provided information on whether validated scales of different aspects of religiosity load together and how single-item measures relate to hypothesised dimensions in a UK birth cohort. These results will help users of the ALSPAC data understand these religiosity variables better. The approach here could also be adopted by other studies relying on single-item religiosity measures to help researchers step back and consider what they are measuring.

## Ethical approval

Ethical approval for the study was obtained from the ALSPAC Ethics and Law Committee (ALEC) and the Local Research Ethics Committee. Informed consent for the use of data collected via questionnaires and clinics was obtained from participants following the recommendations of the ALSPAC Ethics and Law Committee at the time. Informed consent for the use of data collected via questionnaires and clinics was obtained from participants following the recommendations of the ALSPAC Ethics and Law Committee at the time.

## Data Availability

ALSPAC data access is through a system of managed open access. Information about access to ALSPAC data is given on the ALSPAC website ( http://www.bristol.ac.uk/alspac/researchers/access/) and in the ALSPAC data management plan ( http://www.bristol.ac.uk/alspac/researchers/data-access/documents/alspac-data-management-plan.pdf). Data used for this submission will be made available on request to the Executive ( alspac-exec@bristol.ac.uk). The datasets presented in this article are linked to ALSPAC project number B3932, please quote this project number during your application. Please note that the study website contains details of all the data that is available through a fully searchable data dictionary and variable search tool: http://www.bristol.ac.uk/alspac/researchers/our-data/. Study data were collected and managed using REDCap electronic data capture tools hosted at the University of Bristol ^34^. Supplementary information and material is available on the Open Science Framework repository here: https://osf.io/hfgcu/. Open Science Framework (OSF): Dimensionality of Religious Measures Used in ALSPAC. https://doi.org/10.17605/OSF.IO/HFGCU ^35^ This project contains the following underlying data: analysis script.Rmd B3932 Dimensionality of Religious Measures Used in ALSPAC DAP.docx Supplementary.docx Data are available under the terms of the Creative Commons Attribution 4.0 International license (CC-BY 4.0).
