# Supplementary material for: Measuring religiosity in longitudinal secondary datasets: Comparison of single-item measures with validated scales in a UK cohort study (ALSPAC)
Source: Wellcome Open Res. 2025 Sep 22;10:196. Originally published 2025 Apr 18. [Version 2] doi: 10.12688/wellcomeopenres.23943.2 (PMC12519560; doi:10.12688/wellcomeopenres.23943.2)
Supplement: Peer review history

# Reviewer report: Reviewer response for version 2

**DOI:** 10.21956/wellcomeopenres.27488.r133886

**Published:** 2025-10-10

**Authors:** Telli Davoodi

**Affiliations:** 1 Wheelock College of Education & Human Development, Boston University, Boston, USA

I think this looks good now.

Is the work clearly and accurately presented and does it cite the current literature?

Partly

If applicable, is the statistical analysis and its interpretation appropriate?

Partly

Are all the source data underlying the results available to ensure full reproducibility?

Yes

Is the study design appropriate and is the work technically sound?

Partly

Are the conclusions drawn adequately supported by the results?

Partly

Are sufficient details of methods and analysis provided to allow replication by others?

Yes

Reviewer Expertise:

developmental psychology, religious cognition

I confirm that I have read this submission and believe that I have an appropriate level of expertise to confirm that it is of an acceptable scientific standard.

# Reviewer report: Reviewer response for version 2

**DOI:** 10.21956/wellcomeopenres.27488.r135139

**Published:** 2025-10-09

**Authors:** brick johnstone

**Affiliations:** 1 Shepherd Center, Atlanta, Georgia, USA

This study conducted factor analyses to determine how different measures of religiosity cohere with a goal of confirming/identifying unique empirical factors of religiosity. In general, it is suggested that there are four generally accepted religious constructs (i.e., intrinsic, extrinsic, private, public) and several measures from ALSPAC using several samples that are investigated. In general it is concluded that there are various results depending on the type of factor analysis used, with different results indicating different possible solutions: one overall religiosity factor, two factors, and six factors, the latter two which are not completely consistent or clear. It is then concluded that there is no consistent factor structure to represent religiosity in the ALSPAC data, of which future researchers should be aware.

 Although these analyses and conclusions are valid, it is suggested that the authors consider offering some more clear conclusions/recommendations. For example their study seems to suggest that  due to variability in findings, and consistent with much previous research, the factor analyses suggest it may be best for future research using religion variables (and not just for ALSPAC data) consider religiosity  as a unique and distinct construct (suggesting that there may be theoretically different factors, but they are all generally consistent with an overall religion construct). That is, rather than just list their results, it is suggested that they make a recommendation (not just be aware that our study shows different results).

 It may also be helpful to consider other factor analytic studies that investigated different religious versus spiritual constructs and identified distinct religious (one general factor), distinct spiritual (pos spirituality, neg spirituality, forgiveness), and distinct social support (pos congregational support, neg congregational support; Johnstone, B., Yoon, D.P., Franklin, K.L., Schopp, L.H., & Hinkebein, J. (2009). Reconceptualizing the factor structure of the Brief Multidimensional Measure of Religiousness/Spirituality. Journal of Religion and Health, 48 (2), 146-163; Johnstone, B., Bhushan, B., Hanks, R., Yoon, D., & Cohen, D. (2016). Factor Structure of the Brief Multidimensional Measure of Religiousness/Spirituality in US and Indian Samples with Traumatic Brain Injury. *Journal of Religion and Health*, 55, 572-586.). Many of the items they included in their analyses seem to be better conceptualized as measures of spirituality and congregational support than religiosity. Indicating how their results may support these findings may be helpful to future research in this area.

Is the work clearly and accurately presented and does it cite the current literature?

Partly

If applicable, is the statistical analysis and its interpretation appropriate?

Partly

Are all the source data underlying the results available to ensure full reproducibility?

Yes

Is the study design appropriate and is the work technically sound?

Partly

Are the conclusions drawn adequately supported by the results?

Partly

Are sufficient details of methods and analysis provided to allow replication by others?

Yes

Reviewer Expertise:

neuropsychology of transcendence

I confirm that I have read this submission and believe that I have an appropriate level of expertise to confirm that it is of an acceptable scientific standard, however I have significant reservations, as outlined above.

# Reviewer report: Reviewer response for version 1

**DOI:** 10.21956/wellcomeopenres.26414.r122816

**Published:** 2025-05-27

**Authors:** Telli Davoodi

**Affiliations:** 1 Wheelock College of Education & Human Development, Boston University, Boston, USA

The study explores the factor structure of various pre-validated measures and single-item measures of religiosity among a UK sample of children and parents. Although I understand what was done, I'm struggling to understand why. The research is not motivated well and I'm not sure what problem the paper is trying to solve. The authors do try to motivate the analysis by discussing the need for understanding what dimension of religiosity are measured by single items and the need to produce evidence on how measures relate to each other and to study outcomes. But the paper does not actually inform these questions. It does conclude that single-item measures do not neatly map onto any theorized dimension of religiosity, but it leaves it at that.

 I also don't know why this kind of analyses (i.e., taking various measures, entering them in an EFA, and figuring out how they all relate to one another) is needed. I believe that in most studies on the cognitive science of religion and religiosity, individual measures are designed and validated with specific theoretical underpinnings, hypotheses, and predicted associations with specific outcome variables in mind. For example, a certain measure may be designed and validated to understand the role of religious belief and participation in emotional wellbeing; another measure may be designed and validated to understand the role of religious belief and behaviors in parenting. I assume that these two measures will be different precisely because they are meant to tap into different constructs and capture different theoretical associations with the respective outcomes. It would be interesting in this hypothetical scenario, for example, to explore whether the measure designed for the first use (emotional wellbeing) explains any variability in parenting, and if so, do various dimensions emerge that differentially explains variability. This is all to say that although I believe that exploring the structure of constructs/measures is usually informative and an important step in research, it is not very clear to me why it is done in this case and what we are learning from it. 

 Another point I want to highlight is that although the paper cites other work on the demographics of this sample, without extensively reading from other sources, we don't know anything about this sample. Understanding the sample better can help readers draw more accurate conclusions about the findings.

Is the work clearly and accurately presented and does it cite the current literature?

Partly

If applicable, is the statistical analysis and its interpretation appropriate?

Partly

Are all the source data underlying the results available to ensure full reproducibility?

Yes

Is the study design appropriate and is the work technically sound?

Partly

Are the conclusions drawn adequately supported by the results?

Partly

Are sufficient details of methods and analysis provided to allow replication by others?

Yes

Reviewer Expertise:

developmental psychology, religious cognition

I confirm that I have read this submission and believe that I have an appropriate level of expertise to state that I do not consider it to be of an acceptable scientific standard, for reasons outlined above.

# Response

**Published:** 2025-09-09

**Authors:** Jimmy Morgan

**Affiliations:** University of Bristol, UK

The study explores the factor structure of various pre-validated measures and single-item measures of religiosity among a UK sample of children and parents. Although I understand what was done, I'm struggling to understand why. The research is not motivated well and I'm not sure what problem the paper is trying to solve. The authors do try to motivate the analysis by discussing the need for understanding what dimension of religiosity are measured by single items and the need to produce evidence on how measures relate to each other and to study outcomes. But the paper does not actually inform these questions. It does conclude that single-item measures do not neatly map onto any theorized dimension of religiosity, but it leaves it at that.

 Author response: ***Based on the comments from both reviewers, it is clear that the aim of our paper was perhaps not sufficiently articulated. We have now updated the Introduction section to try and make this clearer.*** ***First, we provide additional justification for why understanding the relationships between single-item variables and items from validated scales is relevant (paragraph 4): “This is important information to know, both when designing and interpreting studies using such single-item variables. For instance, say ‘praying when not in trouble’ reflects an intrinsically-religious mindset, while religious attendance is more extrinsically-oriented. In the design phase, researchers can use this information as justification for why ‘religious service attendance’ is a valid proxy for ‘extrinsic religiosity’; conversely, when interpreting results, a relationship with ‘praying when not in trouble’ and another variable could be interpreted with respect to intrinsic religiosity.”*** ***Next, we clarify the aims of the paper (paragraph 5): “Note that the aim of this paper is not to validate scales such as the DUREL and I/EMSS in this sample, but rather to explore how frequently-used single-item measures of religiosity – such as belief in God, religious service attendance, importance of religion/spirituality – ‘hang’  with each other and map on to the hypothesised dimensions such scales intend to measure. By examining how these single-item religiosity variables relate to hypothesised religious constructs, these findings will assist researchers using and interpreting single-item religiosity data, in ALSPAC and perhaps more broadly.”*** ***To better reflect these aims, we have also updated the title from “Measuring religiosity: Comparison of single-item measures with validated scales in a UK cohort study (ALSPAC)” to “Measuring religiosity in longitudinal secondary datasets: Comparison of single-item measures with validated scales in a UK cohort study (ALSPAC)”.*** ***We have also updated the conclusion section to end on the importance of combining the strengths of epidemiological cohort studies with a stronger focus on measurement of key psychological dimensions of religiosity, and how our study plays a small role in nudging the field in this direction: “Epidemiological cohort studies, such as ALSPAC, have many key strengths, including longitudinal data, detailed phenotypic data, and large representative samples: together these make for stronger scientific inferences, particularly with respect to causality. However, a key limitation of such studies is that religiosity – when measured at all – often consists of a small number of single-item measures, often with unknown relations to specific hypothesised psychological dimensions of religiosity. By beginning to probe these relations – albeit in a rather crude and imperfect way given the data available – we hope this will encourage future epidemiological studies to take the measurement of religiosity more seriously, combining the strengths of longitudinal cohort data with precise and theoretically-grounded measurements of religiosity to advance knowledge in this area.”***  

 I also don't know why this kind of analyses (i.e., taking various measures, entering them in an EFA, and figuring out how they all relate to one another) is needed. I believe that in most studies on the cognitive science of religion and religiosity, individual measures are designed and validated with specific theoretical underpinnings, hypotheses, and predicted associations with specific outcome variables in mind. For example, a certain measure may be designed and validated to understand the role of religious belief and participation in emotional wellbeing; another measure may be designed and validated to understand the role of religious belief and behaviors in parenting. I assume that these two measures will be different precisely because they are meant to tap into different constructs and capture different theoretical associations with the respective outcomes. It would be interesting in this hypothetical scenario, for example, to explore whether the measure designed for the first use (emotional wellbeing) explains any variability in parenting, and if so, do various dimensions emerge that differentially explains variability. This is all to say that although I believe that exploring the structure of constructs/measures is usually informative and an important step in research, it is not very clear to me why it is done in this case and what we are learning from it.

 Author response: ***As we have hopefully made clearer above, researchers using ALSPAC religion data (including ourselves) have no control over what measures were available to us as data collection was designed many years ago. To that end, we have used all the applicable measures available in ALSPAC that we believe contributed to an overall view of participant religious and spiritual identity, and examined how these variables relate to the measured items from validated scales (namely intrinsic and extrinsic religiosity from the DUREL and I/EMSS, respectively).*** ***In terms of ‘what we are learning’, we agree that studies should be designed with specific measures in mind; however, as is often the case with secondary data, researchers are beholden to what was collected at the time. To adapt the example given by the reviewer: say a researcher was interested in whether intrinsic and extrinsic religiosity had different relationships with emotional wellbeing, using a secondary dataset. If these direct measures of these constructs were not measured directly, one may wish to use proxies orf these variables, based on the data that is available; say, ‘prayer when not in trouble’ as a proxy for intrinsic religiosity and ‘religious service attendance’ for extrinsic religiosity. Using data from the present study can help inform whether such assumptions are valid (note that they do not seem to be: see Table 4).*** ***By clarifying our study aims , we hope it is now clear why we conducted these analyses and what we can learn from them.***     Another point I want to highlight is that although the paper cites other work on the demographics of this sample, without extensively reading from other sources, we don't know anything about this sample. Understanding the sample better can help readers draw more accurate conclusions about the findings.

 Author response: ***We agree, but unfortunately are restricted by space constraints . However, we have  added a short summary of key demographics to aid readers, while highlighting additional sources for more detailed information (‘Participants’ section of the Methods: “In these samples, all G1 participants were approximately 28 years of age, 48.4% were female, and 47.9% were educated to degree level. The mean age of mothers was 28 (SD = 5.0) at birth of study child, with 12.9% educated to degree level, while the mean age of partners was 29.9 (SD = 6.7) at birth of study child, with 18.2% educated to degree level (for more information on the ALSPAC sample see Boyd et al., 2013; Fraser et al., 2013; Northstone et al., 2019, 2023; Major-Smith et al., 2022 (references 10-14).”***

# Reviewer report: Reviewer response for version 1

**DOI:** 10.21956/wellcomeopenres.26414.r122419

**Published:** 2025-05-13

**Authors:** Tom Clark

**Affiliations:** 1 University of Sheffield, Sheffield, UK

​I'm afraid that I'm struggling to understand the rationale for the study.

 ​T​he paper is very 'exploratory' in that it appears to be a data-driven exercise that hopes to discover the underlying properties that exist across a range of ​items taken from validated scales and individual measures associated with rel​igio​sity. The results detail the findings of 3 exploratory analyses with respect to an established UK birth cohort study - and, as the paper highlights, none of the solutions suggest a particularly convincing factor structure​, although all items are related to religiosity in some way.

 But if that is the purpose of the paper, the results are exactly what you might expect.​ These instruments are designed and developed to measure specific aspects of religion​ (belief, practice, attendance etc), and they are done so with theoretical purpose. Some of these purposes will cross, others will not. Hence you would expect there to be a lot of covariance across ​different items and instruments, ​a​nd even more when you add single measures.​ It is this 'noise' that is being picked up by the EFA​ results.

 ​T​his is especially the case as the authors of DUREL note that the (5 item) instrument consists of 3 subscales and do not recommend using it as a single scale​. Instead, they suggest entering the subscales into a regression analysis separately as they can (and do) operate in different ways. 

 ​That said, other authors have validated DUREL using a single factor structure. However, these studies have used the conventional approach of implementing EFA followed by CFA to validate the scale. Indeed, it would be conventional to only use the ​o​riginal items from the scale to do this, ​u​nless there was an explicit theoretical justification to do otherwise. Without that justification, it begins to look like an answer in search of a question.

 If you still *really* wanted to include everything, it is probably possible to account for ​some of this covariance using SEM - the lavaan package in R has recently introduced the capacity for ESEM for instance - but the model would be complex and lack parsimony.​ Exploratory Graph Analysis could also be used to examine the relationships in more detail.

 So, what to do about it?

 ​In this case, there is a data analysis plan document in the supplementary materials that outlines the intention​ of the paper from the outset. This provides some additional detail in respect to the rationale for the paper, and can serve as a pre-registration document by proxy. Therefore - and although I'm pushing open science to its limits here - there is a reason to publish the results.   

 ​However, I would expect to see a more conventional analysis of the DUREL in the first instance using EFA/CFA. This would provide a stronger rationale for the use of wider measures (and if it demonstrates good fit, you can use the scale to examine how additional items might relate to it). 

 While the format of the 'research note' does not lend itself to lengthy methodological discussion, It would also be conventional to have some theoretical justification for the instruments included in the study. Currently, this justification is a little haphazard (and by the end, it feels as though the authors are disowning it a little), but there is more detail in the data analysis plan that could be adapted. This would lead to a more defensible position concerning what you were expecting to find, and why those measures were chosen in particular.

Is the work clearly and accurately presented and does it cite the current literature?

Partly

If applicable, is the statistical analysis and its interpretation appropriate?

No

Are all the source data underlying the results available to ensure full reproducibility?

Yes

Is the study design appropriate and is the work technically sound?

No

Are the conclusions drawn adequately supported by the results?

Partly

Are sufficient details of methods and analysis provided to allow replication by others?

Yes

Reviewer Expertise:

Research methods, including survey design and instrument development.

I confirm that I have read this submission and believe that I have an appropriate level of expertise to confirm that it is of an acceptable scientific standard, however I have significant reservations, as outlined above.

# Response

**Published:** 2025-09-09

**Authors:** Jimmy Morgan

**Affiliations:** University of Bristol, UK

​I'm afraid that I'm struggling to understand the rationale for the study.

 ​T​he paper is very 'exploratory' in that it appears to be a data-driven exercise that hopes to discover the underlying properties that exist across a range of ​items taken from validated scales and individual measures associated with rel​igio​sity. The results detail the findings of 3 exploratory analyses with respect to an established UK birth cohort study - and, as the paper highlights, none of the solutions suggest a particularly convincing factor structure​, although all items are related to religiosity in some way.

 Author response: ***We thank the reviewer for their timely and constructive response. They have made numerous suggestions on how to improve the paper and we have endeavoured to address them below. We agree, the paper is very exploratory, as the aim was to see how all of our secondary data, with different religious dimensions measured was related to each other. We wanted to do this to determine whether these multiple measures could be reduced into simple patterns for future research use. We have taken a number of steps to make the exploratory nature of the study, as well as the rationale for this exploration clearer, as will be seen in the responses below.*** ***We have updated the Introduction section to clarify the rationale.*** ***First, we provide additional justification for why understanding the relationships between single-item variables and items from validated scales is relevant (paragraph 3): “This is important information to know, both when designing and interpreting studies using such single-item variables. For instance, say ‘praying when not in trouble’ reflects an intrinsically-religious mindset, while religious attendance is more extrinsically-oriented. In the design phase, researchers can use this information as justification for why ‘religious service attendance’ is a valid proxy for ‘extrinsic religiosity’; conversely, when interpreting results, a relationship with ‘praying when not in trouble’ and another variable could be interpreted with respect to intrinsic religiosity.”*** ***Next, we clarify the aims of the paper (paragraph 4): “Note that the aim of this paper is not to validate scales such as the DUREL and I/EMSS in this sample, but rather to explore how frequently-used single-item measures of religiosity – such as belief in God, religious service attendance, importance of religion/spirituality – ‘hang’  with each other and map on to the hypothesised dimensions such scales intend to measure. By examining how these single-item religiosity variables relate to hypothesised religious constructs, these findings will assist researchers using and interpreting single-item religiosity data, in ALSPAC and perhaps more broadly.”*** ***To better reflect these aims, we have also updated the title from “Measuring religiosity: Comparison of single-item measures with validated scales in a UK cohort study (ALSPAC)” to “Measuring religiosity in longitudinal secondary datasets: Comparison of single-item measures with validated scales in a UK cohort study (ALSPAC)”.*** ***Finally, we have updated the conclusion section to end on the importance of combining the strengths of epidemiological cohort studies with a stronger focus on measurement of key psychological dimensions of religiosity, and how our study plays a small role in nudging the field in this direction: “Epidemiological cohort studies, such as ALSPAC, have many key strengths, including longitudinal data, detailed phenotypic data, and large representative samples: together these make for stronger scientific inferences, particularly with respect to causality. However, a key limitation of such studies is that religiosity – when measured at all – often consists of a small number of single-item measures, often with unknown relations to specific hypothesised psychological dimensions of religiosity. By beginning to probe these relations – albeit in a rather crude and imperfect way given the data available – we hope this will encourage future epidemiological studies to take the measurement of religiosity more seriously, combining the strengths of longitudinal cohort data with precise and theoretically-grounded measurements of religiosity to advance knowledge in this area.”***

 But if that is the purpose of the paper, the results are exactly what you might expect.​ These instruments are designed and developed to measure specific aspects of religion​ (belief, practice, attendance etc), and they are done so with theoretical purpose. Some of these purposes will cross, others will not. Hence you would expect there to be a lot of covariance across ​different items and instruments, ​a​nd even more when you add single measures.​ It is this 'noise' that is being picked up by the EFA​ results.

 Author response: ***Whilst the primary purpose of the paper was to explore how the religious measures in ALSPAC might relate to one another, as discussed above, we also believed there was potential to confirm that measures would relate to pre-hypothesised dimensions  in combination and that this in itself would be a useful outcome.  ALSPAC included the DUREL and I/EMSS at later timepoints with the aim of creating a more robust idea of participant religiosity for future investigation. However, the initial measures included in the first 4 (of 5) timepoints were much less comprehensive and related less to different dimensions of religion and were more intended to give an idea of participant religiosity as part of a broader overview of the participants’ demographics and potential confounders of other health outcomes. It is with this in mind that we have conducted this study into how these measures of religiosity compare with more purpose-built regimens of religious variables to inform the use of our variables.***

 ​T​his is especially the case as the authors of DUREL note that the (5 item) instrument consists of 3 subscales and do not recommend using it as a single scale​. Instead, they suggest entering the subscales into a regression analysis separately as they can (and do) operate in different ways.  That said, other authors have validated DUREL using a single factor structure. However, these studies have used the conventional approach of implementing EFA followed by CFA to validate the scale. Indeed, it would be conventional to only use the ​original items from the scale to do this, ​unless there was an explicit theoretical justification to do otherwise. Without that justification, it begins to look like an answer in search of a question.

 Author response: ***We agree. However, we did not use the DUREL items as a single scale. Instead, we took each of the 5 individual DUREL items , and used these in our analyses. As discussed above, the aim of the present study was to attempt to assess the disparate measures in ALSPAC and provide advice as to how they may be used in the future; not to specifically validate the factor structure of, for example, the DUREL. This should now be clear in the introduction.*** ***As epidemiology studies often have large-scale longitudinal data, but pay less attention to measures, we are attempting to examine the validity of these pre-existing measures, which can be found in many longitudinal secondary datasets.*** ***We have also updated our language in a few places where this may have been unclear. E.g., from “confirm whether the validated measures work as intended” to “assess whether the validated measures load together as expected” (abstract).***

 If you still *really* wanted to include everything, it is probably possible to account for ​some of this covariance using SEM - the lavaan package in R has recently introduced the capacity for ESEM for instance - but the model would be complex and lack parsimony.​ Exploratory Graph Analysis could also be used to examine the relationships in more detail.

 Author response: ***Thank you for this suggestion. However, as the aim of our analyses is simply to see how these religiosity variables ‘hang together’ - as opposed to specifically validate certain religiosity scales such as the DUREL - we believe that our analyses are appropriate for our aims.***

 So, what to do about it?

 ​In this case, there is a data analysis plan document in the supplementary materials that outlines the intention​ of the paper from the outset. This provides some additional detail in respect to the rationale for the paper, and can serve as a pre-registration document by proxy. Therefore - and although I'm pushing open science to its limits here - there is a reason to publish the results.   

 Author response: ***We appreciate the reviewer noting our published analysis plan as a strength. We do note, however, that after analysing and exploring the data we decided to split that analysis plan  into two papers: one on how the single-item variables map onto hypothesised dimensions of religiosity (the present study), and another on dimension reduction of religiosity variables when using ALSPAC religion data, which is in preparation. We have added a paragraph at the end of the analysis section of the methods explaining that this is the first of two papers based on the analysis plan: “This paper is based on a pre-published analysis plan available on the Open Science Framework here: https://osf.io/hfgcu/ . We made the decision to split this analysis plan into two separate papers due to the difference in aims, this is the first of said papers.”***

 ​However, I would expect to see a more conventional analysis of the DUREL in the first instance using EFA/CFA. This would provide a stronger rationale for the use of wider measures (and if it demonstrates good fit, you can use the scale to examine how additional items might relate to it). 

 Author response: ***Again, while we appreciate the reviewers suggestion, the aim of our study is not to validate religiosity scales such as the DUREL. This is us trying to make the most of existing measures, which often consist of chopped and changed versions of full psychological measures (e.g., although we possess all five questions of the DUREL, we only possess two measures of extrinsic religiosity from the I/EMSS). This is especially the case in epidemiology where single items and binary responses are often used in place of fully-validated scales.***

 While the format of the 'research note' does not lend itself to lengthy methodological discussion, It would also be conventional to have some theoretical justification for the instruments included in the study. Currently, this justification is a little haphazard (and by the end, it feels as though the authors are disowning it a little), but there is more detail in the data analysis plan that could be adapted. This would lead to a more defensible position concerning what you were expecting to find, and why those measures were chosen in particular.

 Author response: ***As the measures used in this study were not chosen by us it is difficult to attempt to justify the ones used for any principled reason other than availability. As the reviewer mentions it has been difficult to fit in enough theoretical justification for the paper within the confines of a research note. We have however, added more justification to the introduction outlined above.***
